# Dual-band MIMO antenna with low mutual coupling for 2.4/5.8 GHz communication and wearable technologies

**DOI:** 10.1371/journal.pone.0301924

**Published:** 2024-04-17

**Authors:** Wahaj Abbas Awan, Tanvir Islam, Fahad N. Alsunaydih, Fahd Alsaleem, Khaled Alhassoonc

**Affiliations:** 1 Department of Information and Communication Engineering, Chungbuk National University, Cheongju, South Korea; 2 Department of Electrical and Computer Engineering, University of Houston, Houston, TX, United States of America; 3 Department of Electrical Engineering, College of Engineering, Qassim University, Unaizah, Saudi Arabia; Bahria University, PAKISTAN

## Abstract

To satisfy the requirements of modern communication systems and wearables using 2.4/5.8 GHz band this paper presents a simple, compact, and dual-band solution. The antenna is extracted from a circular monopole by inserting various patches and stubs. The genetic algorithm is utilized to optimize the parameters and achieve the best possible results regarding bandwidth and gain. Afterward, a 2-port multiple-input-multiple-output (MIMO) configuration is created by positioning an identical second single element perpendicularly to the first one. The electrical size of the suggested MIMO configuration is 0.26 λ_L_ × 0.53 λ_L_, where λ_L_ represents the free space wavelength at lower resonance of 2.45 GHz. The common ground technique is adopted to further reduce and achieve the accepted level of mutual coupling of the MIMO configuration. The presented MIMO antenna offers a low mutual coupling of < –27 dB with 0.2 envelope correlation coefficient (ECC). The antenna has a gain of around 6.2 dBi and 6.5 dBi at resonating frequencies of 2.45 GHz and 5.4 GHz. Furthermore, the specific absorption rate (SAR) analysis of the MIMO antenna offers a range inside of the standard values, showing its potential for On/Off body communications. The comparison with already published works shows that the proposed antenna achieves better results in either compact size or wide operational bandwidth along with low mutual coupling.

## Introduction

Antenna is a pivotal component in the front end of advanced wireless communication. This growth in demand for wireless communication requires developing and modifying the design of an antenna [1, 2]. A compact, simple, and low-profile antenna is required for current and upcoming 5G and 6G communication systems, along with high antenna gain, wideband, low mutual coupling, and other basic and advanced parameters of the antenna [3–5]. Moreover, the increased demand for wearables also makes it critically important that antennas work properly around the human body [6]. A compact and simplified geometry antenna operating over the industrial, scientific and medical (ISM) and wireless local area networks (WLAN) bands has remained a top research topic during the past decade. The conversion of these simple geometric structures into multi-input-multi-out (MIMO) systems is also a trending research topic among academia and researchers [7–9]. The most recent development in MIMO antennas is an effort in order to refine the functionality of the antenna by introducing various techniques. The outcomes of MIMO antennas are improved by loading electronic band gap (EBG) structures, defected ground structure (DGS), metamaterials, frequency selective surfaces (FSS), and parasitic patches between antenna elements [10–12]. It should be mentioned that EBG structures can also be used for a variety of applications, such as, spatial filters [13–15] and photonic crystals [16–18]. Moreover, they have a lot of applications in microstrip components [19–21]. Mutual coupling is a primary and important specification of MIMO antenna systems [22, 23]. Mutual coupling reduction of antenna elements can be performed in a number of ways such as the insertion of parasitic patches and the DGS ground plane.

It is mentioned in [24] an antenna offering 2.4 and 5.2 GHz with 2.4–2.63 GHz and 5.13–5.8 GHz bandwidths and has a tiny footprint of 25.7 mm × 22 mm × 0.8 mm. Despite the antenna’s small size, straightforward shape, and good bandwidth, it has a modest peak gain. According to [25], a simpler geomagnetic antenna is used for ISM applications. The antenna employs the 2.43–2.48 GHz frequency band and has enormous dimensions of 38.2 mm by 95.94 mm by 1.6 mm. Work pertaining to ISM and WLAN applications is addressed in [26]. The antenna has a good bandwidth of 2.34–2.5/5.06–5.91 GHz at the operating frequency bands of 2.4 GHz and 5.4 GHz. The drawback of this work is its complicated geometry and lack of gain data. A wide-slot double-frequency antenna for ISM and WLAN purposes is provided in [27]. The antenna has a high gain of 4.89/5.75 over the 2.45 and 5.1 GHz operational frequencies, with a 2.4–2.6/4.95–5.3 GHz bandwidth. Although the proposed solution is high gain and broadband, it has large dimensions, making it unsuitable for compact antennas. 2.4 GHz and 5.8 GHz are the planned frequency bands for this effort, according to [28]. Artificial magnetic conductor (AMC) metamaterial is loaded into the reported work to achieve a high maximum gain of 4.92 dBi and 4.76 dBi. The drawback of this work is that it operates at operational frequencies of 2.4 GHz and 5.8 GHz, respectively, with complicated geometries and a limited operating bandwidth of 240 MHz. The work reported in [29] consists of simplified geometry consisting of a rectangular patch with two pair of rectangular slits and a moderate gain of > 2 dBi at the upper band along with that the antenna offers narrow bandwidth at both resonances and had a setback of large size.

To overcome the aforementioned requirements, a number of MIMO antennas were proposed to generate a high data rate and capacity solution for use in 5G applications [30]. Several methods, such as loading parasitic patches, decouplers, DGS, or dielectric resonators are proposed to reduce the mutual coupling of MIMO antennas [30–41]. In [31], a compact 4-element MIMO antenna is proposed for ultra-wideband applications. The mutual coupling of the antenna is enhanced to a minimum of -17 dB by using DGS. Another work on isolation improvement is given in [32] for ISM and WLAN applications. The antenna has a wide bandwidth of 2.1–2.7 GHz and 5.1–6.1 GHz at operating frequencies respectively at 2.5 GHz and 5.4 GHz bands. The disadvantages of the afore-mentioned design are complex geometry, a lower value for minimum isolation, and a lack of study of MIMO parameters. A two-port MIMO antenna offering frequency bands of 2.4, 3.5, and 5.5 GHz with respective bands of 2.29–2.47 GHz, 3.34–3.73 GHz, and 4.57–6.75 GHz is reported in [33]. The given design offers wideband and high gain but has the drawback of low mutual coupling. Besides using a slotted ground approach, the work offers a mutual coupling of -22 dB. The isolation of antennas can be improved by using meta-surface and shorting pins. A four-element MIMO antenna with increased isolation is described in [34] and uses shorting pins and slots. The reported antenna has an 8 dBi gain with big dimensions of 46 mm × 46 mm × 1.52 mm, but it offers minimum isolation of 32 dB. For ISM and WLAN implementations, a two-band simple monopole is given in [35]. The overall antenna occupies a size of 45 mm by 45 mm by 0.4 mm and provides a minimum isolation of 20 dB.

A four-element MIMO antenna with enhanced isolation for ISM applications [36] has a wide operational frequency band (2.16–3.2 GHz), with isolation 21 dB. The drawbacks of this design are its complex shape, large size, and low peak gain of <1.2 dBi. An excellent gain antenna with circular polarization is reported in [37] for vehicle-to-everything (V2X) communication. The antenna offers a narrow bandwidth of 5.8–5.9 GHz with a high maximum gain of 8.72 dBi. Also, it has a minimum mutual coupling value of –25 dB, and a parasitic patch along with DGS is introduced to obtain these results. A closely-packed antenna operating in a narrow band of 2.5–2.55 GHz and with measurements of 100 × 100 × 0.8 mm^3^ is disclosed in [38]. Besides these works, a number of other works for various wireless applications are reported in [39–41] with improved isolation by loading parasitic patches. It is evident from the foregoing discussion and comparison with the literature that more study is needed to create an antenna with a simple geometry, compact in size, offering high gain as well as efficiency, and providing good values for other MIMO parameters, especially mutual coupling and envelope correlation coefficient (ECC).

This manuscript recommends an antenna for dual-band ISM and WLAN applications. The antenna has simplified geometry, a stub-loaded patch and co-planar waveguide (CPW) feedline, and a compact size. It offers a high gain and also better results for other relevant parameters of MIMO antennas. The novel approach is used to refine the mutual coupling of the suggested work. Section II discusses a single antenna element, compares its findings to the literature, and contrasts simulated and measured results. The two MIMO antennas with refined isolation are examined in Section III. Section IV summarizes the recommended work and provides literature references.

## Antenna design and optimization

This section analyzes a single element of the recommended work accompanying the fabricated prototype. The outcomes in terms of |S11|, frequency vs gain, radiation efficiency, and pattern of radiation are discussed by comparing predicted results by software HFSSv9 and measured results. In the end, the results of the recommended single-element antenna are compared with the literature as well. Table 1 summarized the optimized dimensions of the proposed antenna.

**Table 1:** Proposed antenna dimensions in detail.

Parameters	Values (mm)	Parameters	Values (mm)
W_1_	18	L_1_	33
W_2_	12	L_2_	10
W_3_	8	L_3_	3
W_4_	10	L_4_	1.5
W_5_	7	L_5_	2
L_f_	12.6	L_6_	4.5
W_f_	2	R_0_	4
cu	0.035	H	1.6

### Antenna design

**Fig 1:**
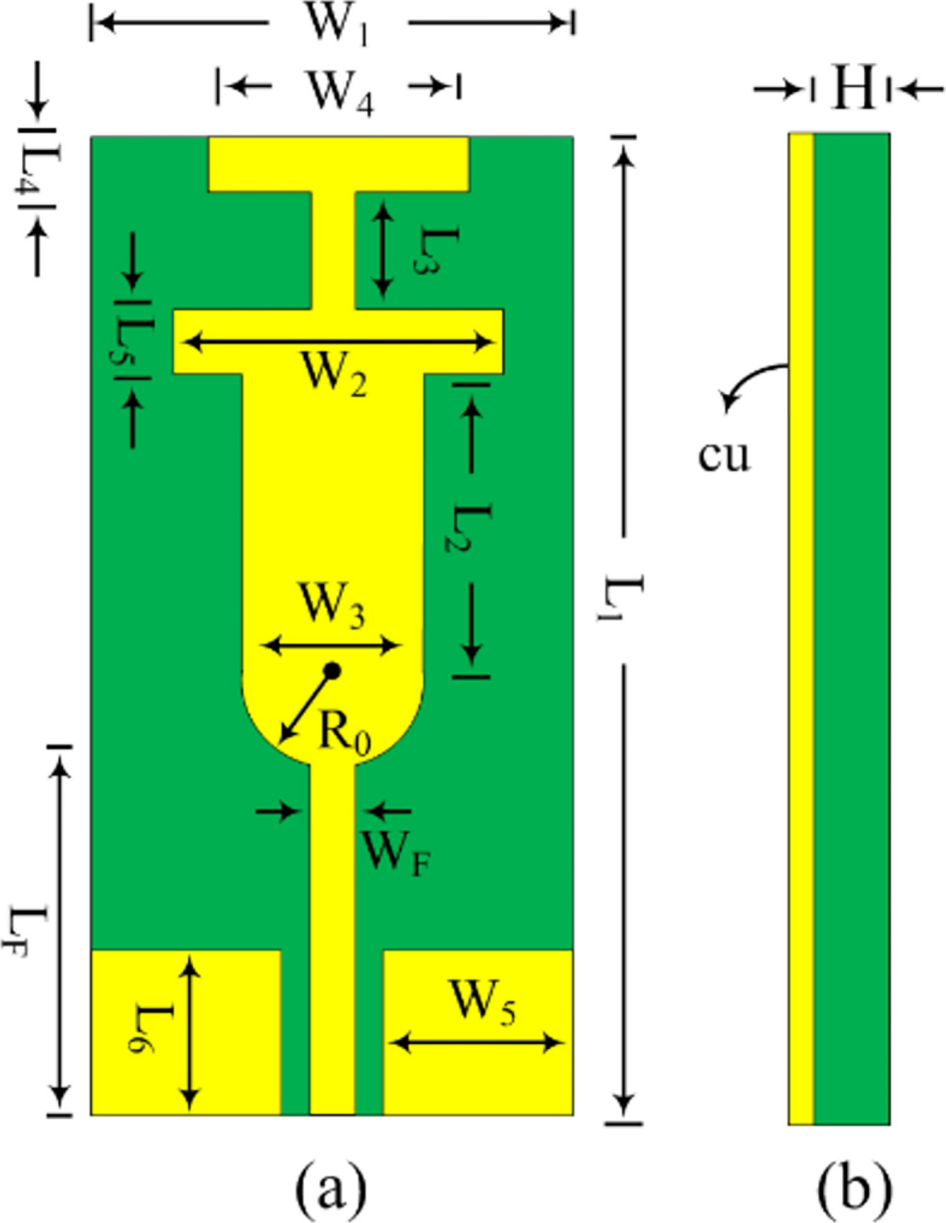
Design configuration of the suggested single antenna (a) Front layout (b) Side layout.

The suggested dual-band antenna operating in the ISM and WLAN frequencies is shown in Fig 1 by its structural layout. The proposed antenna is clearly made up of a CPW feedline line packed with several stubs. Many stubs are loaded to obtain the broad and appropriate resonance frequencies. The planned work is fabricated on Roger RT/Duroid 5880 and has a height of 0.79 mm and a relative permittivity of 2.2. ANSYS HFSS was utilized as an EM simulator to assess the design of the suggested antenna. After that, a real antenna was constructed to validate the simulation’s findings.

### Design methodology

**Fig 2:**
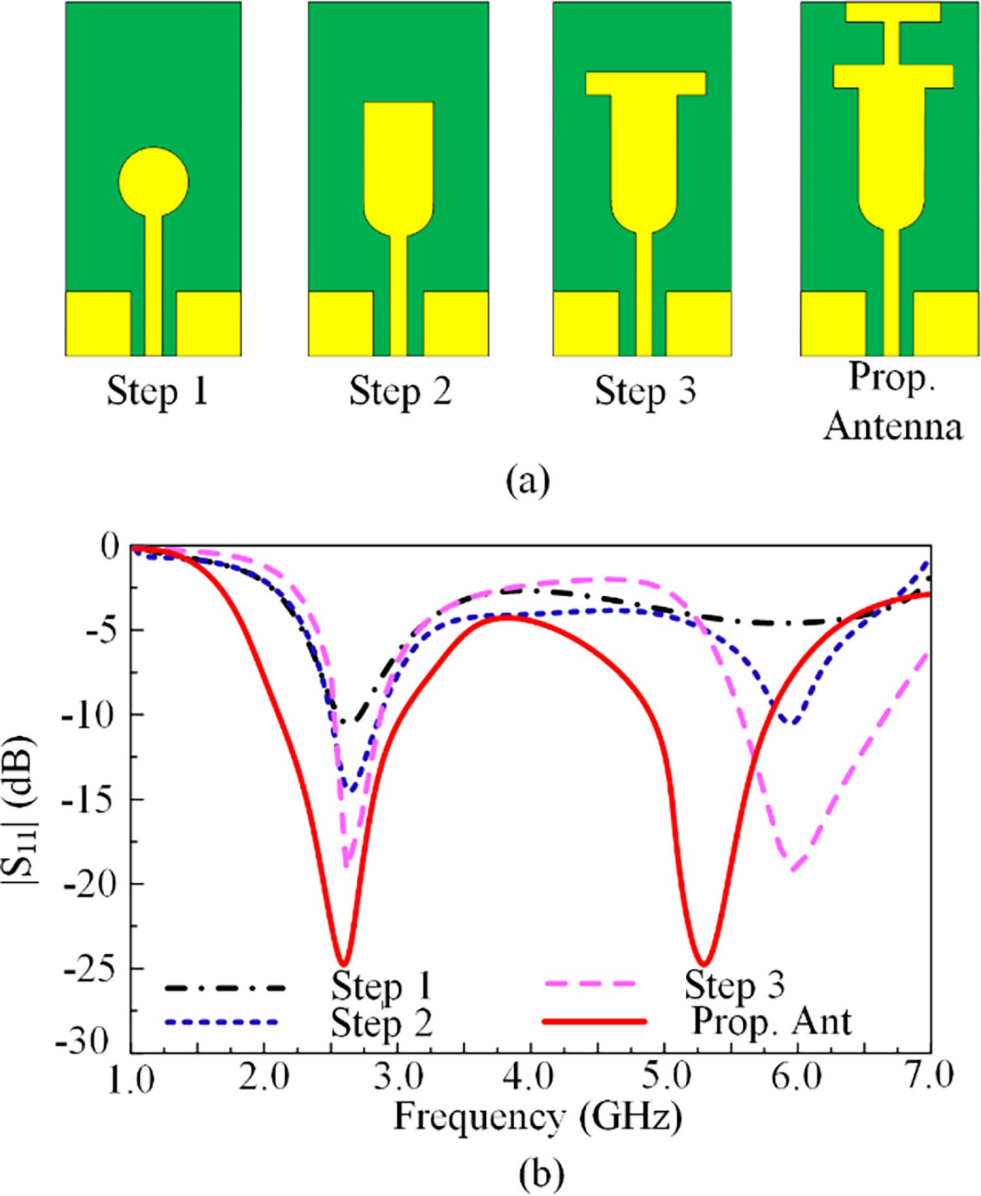
(a) Design stages of the recommended antenna (b) along with variation in |S11| vs Frequency plot.

The outcomes of the proposed work are attained after completing various design processes. For 2.45 GHz ISM applications, a circular-shaped patch with R_0_ = 4 mm, as shown in Fig 2(A), is constructed in the first stage, whose dimensions can be extracted using the expression provided in [8]. The antenna has a reflection coefficient of -10dB and operates over 2.5 GHz, as shown in Fig 2(B). As mentioned in Fig 2(A), a rectangular stub is inserted to reduce mismatching and increase bandwidth. This step improves the reflection coefficient at 2.5 GHz from—10 dB to—15 dB and an extra band is gained around 6 GHz. In the third phase, a rectangular stub with a width W2 of 10 mm is introduced onto the top of the radiator. This step of the design results in a two-band antenna operating at 2.6 and 6 GHz offering bandwidths of 2.3–2.9 GHz and 5.6–6.75 GHz, respectively. As given in Fig 2(B), the antenna offers a wideband behavior in each of its bands. In order to obtain the desired bands, the T-shaped stub is implemented on upper side of the existing stub-loaded patch radiator. The final geometry is obtained, and the antenna impedance experiences a shift towards lower band. At this stage, the antenna operates at two operating frequency bands 2.45 GHz and 5.4 GHz.

### Parametric analysis

**Fig 3:**
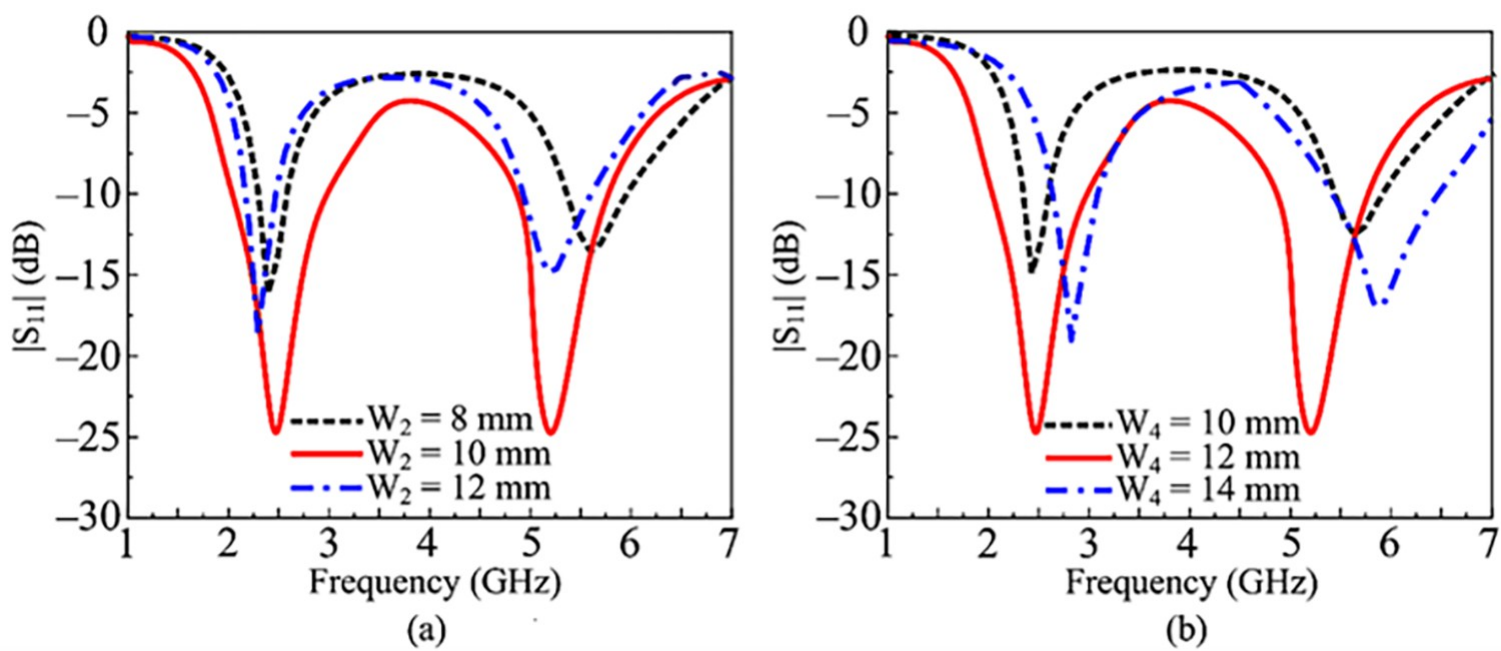
Parametric analysis of key parameters of the recommended antenna (a) Effect by variation in top-loaded stub W4 (b) Effect by variation in lower stub W2.

The parametric analysis of key parameters is carried out to study the effects of each parameter in obtaining the wideband characteristic. Fig 3(A) shows the variation in |S11| parameter by varying the width of the lower stub, W2. It delivers dual-band behavior in the 2–3 GHz range and 4.8–6.1 GHz at its set point of 12 mm. The reflection coefficient of the antenna is decreased to -15 dB, which also affects bandwidth, if the value is raised to 14 mm. The impact on reflection coefficient and bandwidth is additionally apparent in Fig 3(A) if the W2 is decreased to 10 mm. Fig 3(B), illustrates how changing W4, the top stub’s width, impacts |S11|. It delivers a dual-band reflection coefficient response with the abovementioned broad bandwidth at the optimum value of 10 mm. The antenna provides a narrowband behavior and is unable to operate in the required frequency bands if W4 is kept at 8 mm. The antenna’s working frequencies are 3 GHz and 6.5 GHz, respectively, with bandwidths between 2.7–3.2 GHz and 6–7 GHz, if the value is higher than the ideal value of 12 mm.

### Design optimization

**Fig 4:**
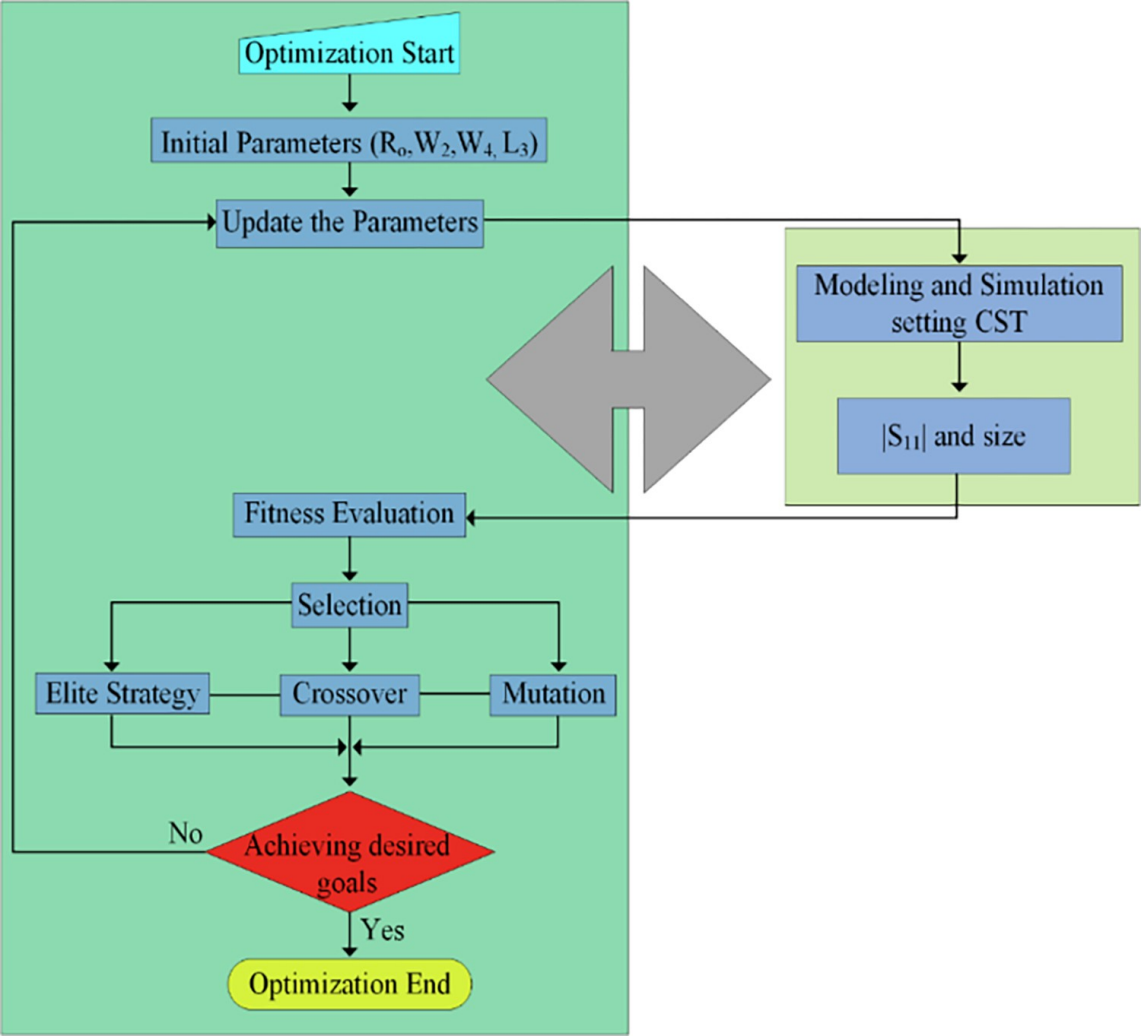
Optimization algorithm flowchart.

Fig 4 represents the flow chart, which shows the design optimization steps of the suggested antenna. Initially, a microstrip patch antenna with a circular-shaped slot with a radius of R0 is designed. The suggested antenna’s dimensional measurement is adjusted to obtain the desired outcomes. The basic circular shape is remodeled by loading regular stub to improve the bandwidth and impedance matching. The length and width of rectangular stubs are adjusted at their best outcomes, examined by studying parametric analysis. After that, two stubs are positioned at the top of the patch having widths of W2 and W4. The optimization of the width and length of these two stubs gets the optimum value of the parameters to attain the maximal bandwidth. The parameters W2, W4 and L3 are analyzed to get the outstanding outcomes in terms of bandwidth. It should be mentioned that other artificial intelligence algorithms, such as particle swarm optimization [42], multi-agent system [43, 44], genetic algorithm [45], and grey wolf optimization [46] can be adopted for further optimization.

### Dual-band antenna measurements and results

**Fig 5:**
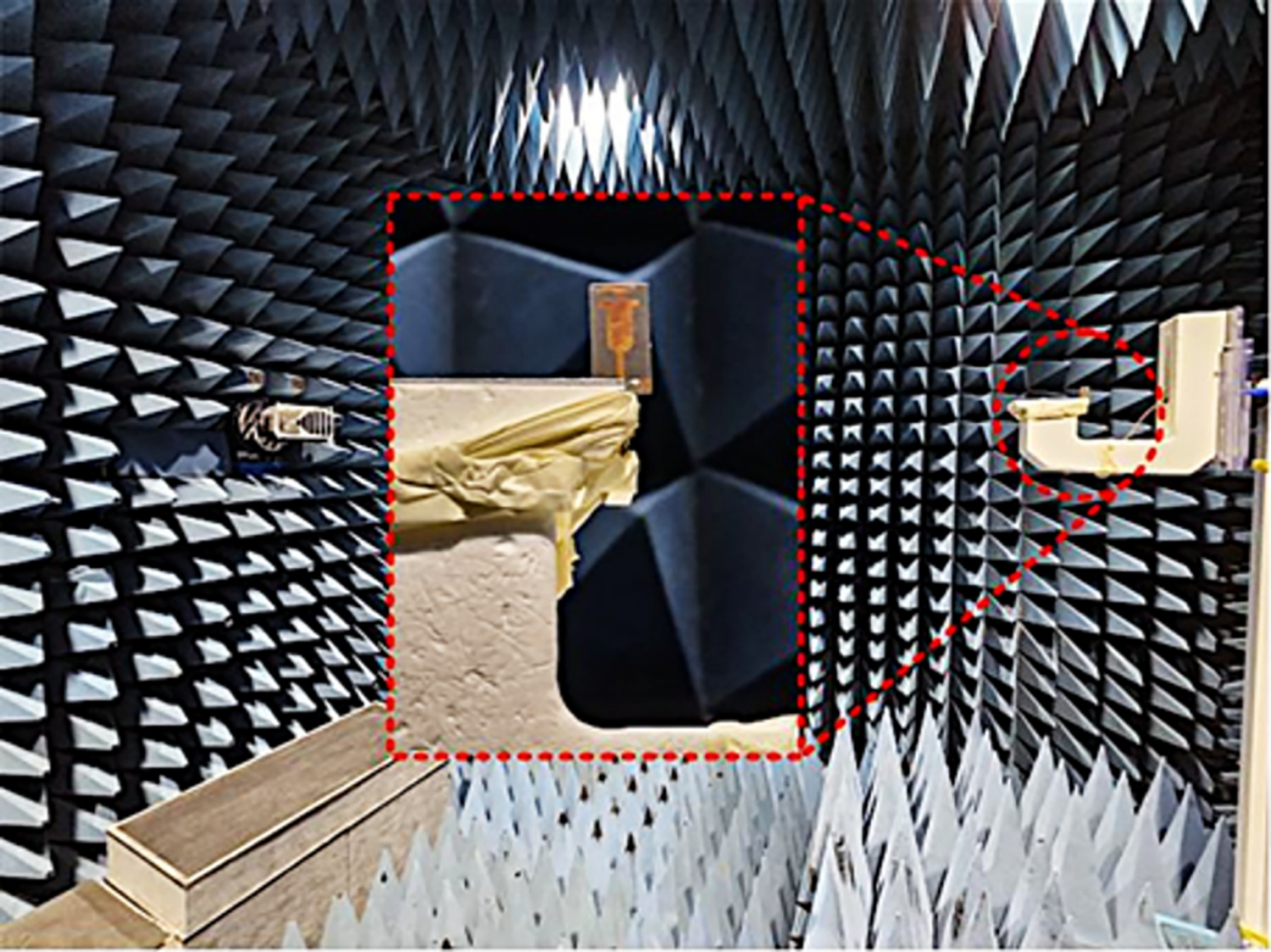
Recommended single-element antenna’s measurement setup.

To examine the outcomes of the antenna, various key parameters are studied. In order to verify these simulated results, a hardware model of the designed antenna is fabricated to measure the results and compare them with simulated ones, as shown in Fig 5.

### S-parameters

**Fig 6:**
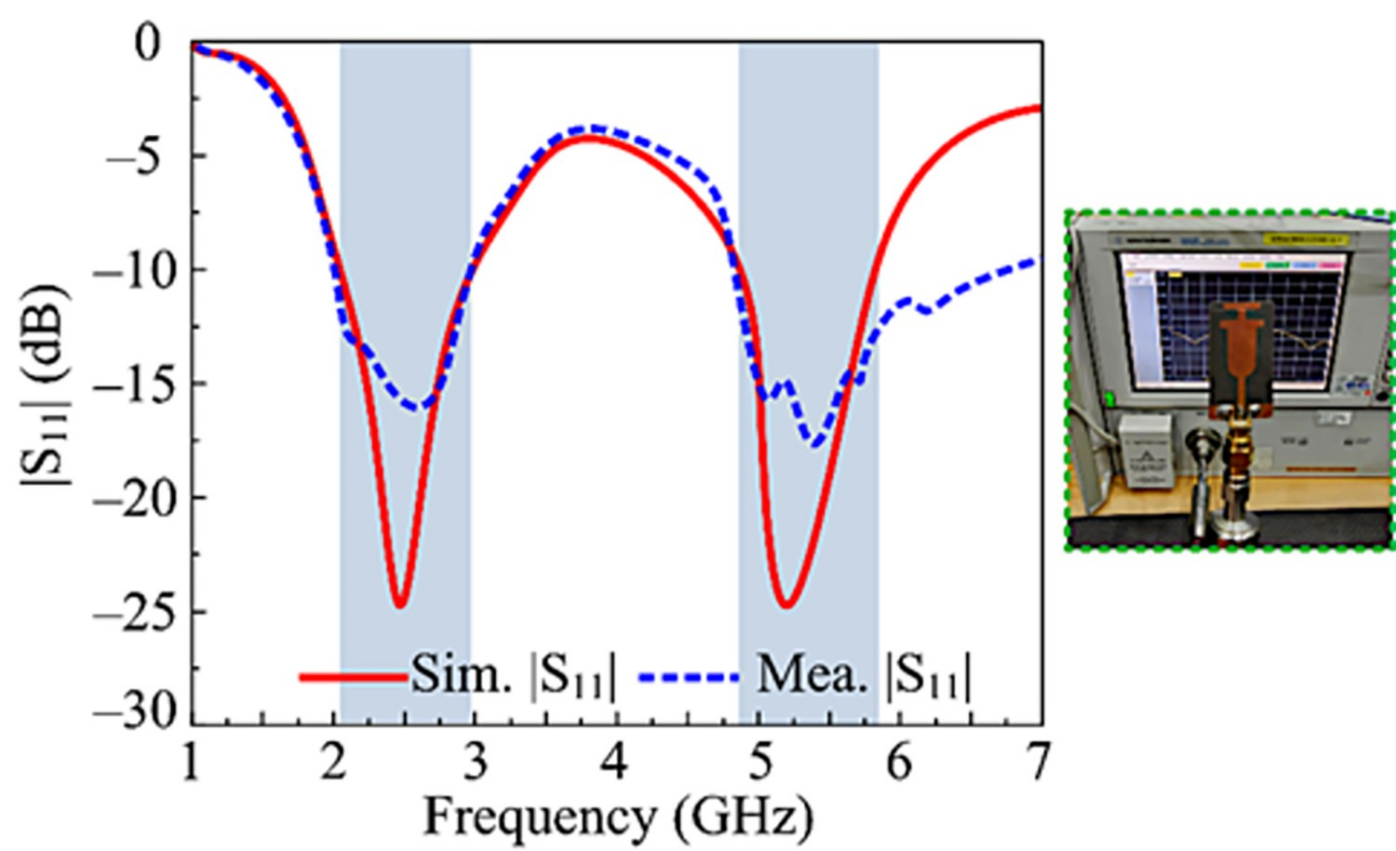
Predicated and measured |S11| parameter of the recommended antenna along with fabricated prototype.

Fig 6 shows the simulated and measured |S11| parameter of the proposed solution. The figure represents that the operational impedance bandwidth is between 2.1–2.95 GHz and 5.12–5.9 GHz. Additionally, at 2.45 and 5.4 GHz, the recommended work gives a re-flection coefficient of -24 dB. Also, the hardware measurements and software findings exhibit remarkable similarities, making the proposed architecture the best candidate for future devices that operate over the ISM and WLAN frequency ranges.

### Radiation pattern

**Fig 7:**
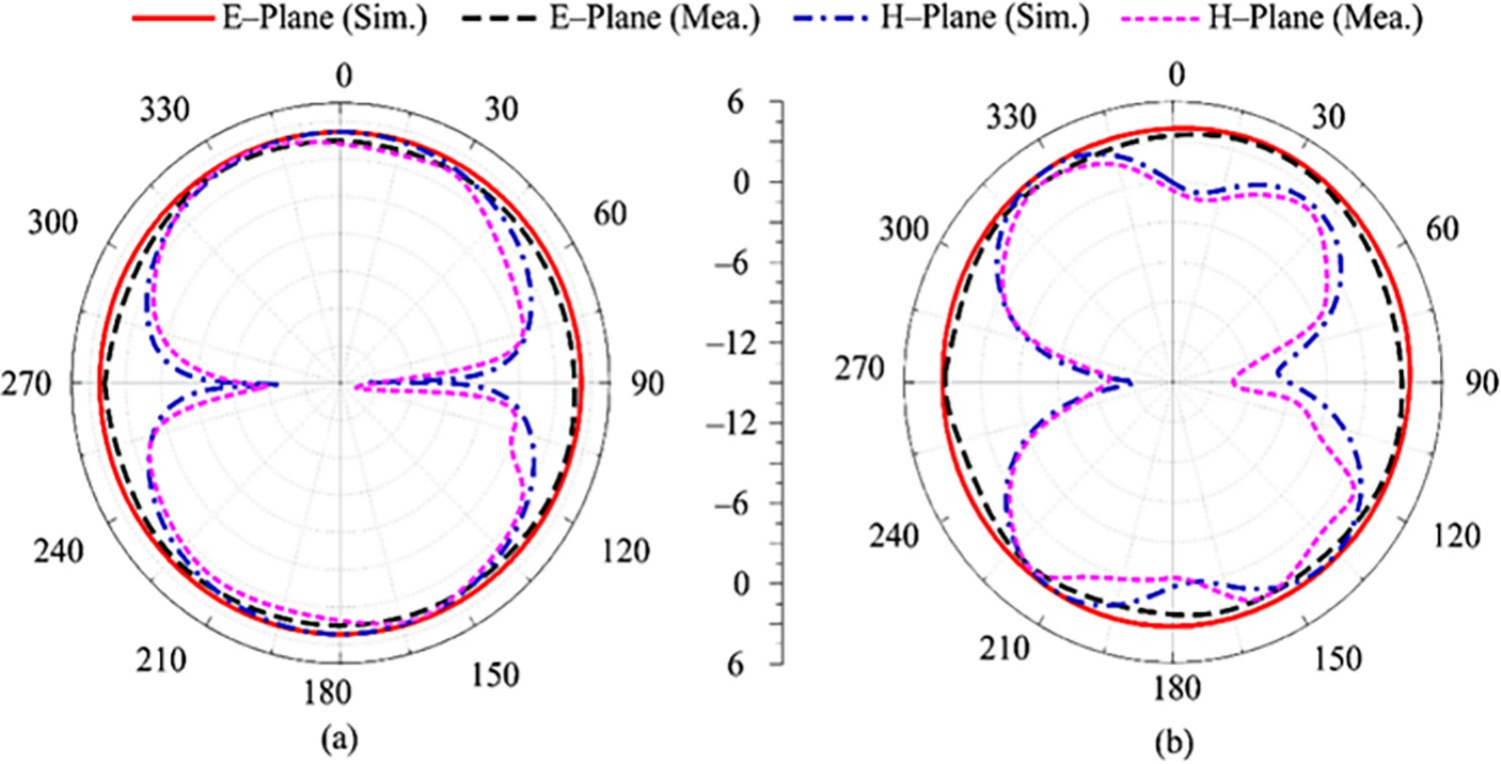
Predicated and measured radiation pattern characteristics of the recommended antenna at (a) 2.45 GHz (b) 5.4 GHz.

Another crucial factor to consider while evaluating an antenna’s performance is its radiation pattern. The radiation pattern at 2.45 GHz and 5.2 GHz is given in Fig 7(A) and 7(B), respectively. It can be shown that the recommended work gives omnidirectional radiation in the E-plane at 2.45 GHz and bidirectional radiation in the H-plane. For 5.4 GHz, the E–plane pattern is same as 2.45 GHz, which is omni–directional but for the H–plane the pattern shows small distortion while maintaining a bi-directional pattern. This distortion is due to the multi–stub loading and shifting towards higher frequency. Moreover, the measured radiation pattern and simulated results show excellent coherence. The correlation in tested and simulated findings and radiation pattern analysis makes the recommended antenna a suitable solution for wireless devices operating over 2.45 GHz and 5.4 GHz.

### Gain vs frequency plot

**Fig 8:**
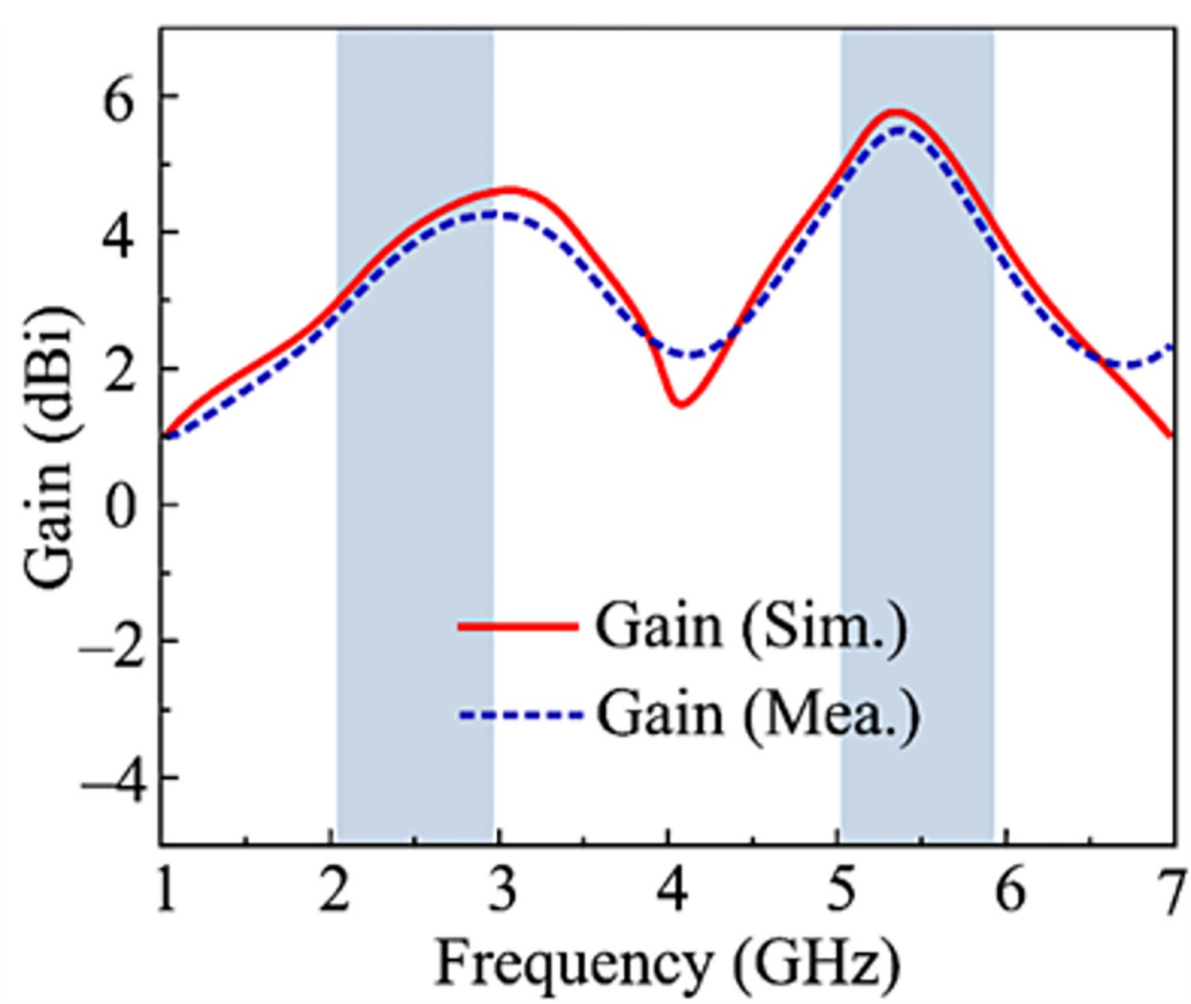
Predicated and tested gain over frequency plot of recommended design.

The simulated and measured gain of the recommended configuration is illustrated in Fig 8. The antenna delivers a gain of >3 dBi across the operational frequency range of 2.1–2.95 GHz, with the value of 4 dBi at 2.45 GHz. On the other hand, the antenna pro-vides gain >4.25 dBi at the 5.1–5.9 GHz working bandwidth with an intensity of 5.2 dBi at the 5.4 GHz. The comparison shown in Fig 8 also makes it evident that the measured and simulated results offer a high similarity.

### Radiation efficiency

**Fig 9:**
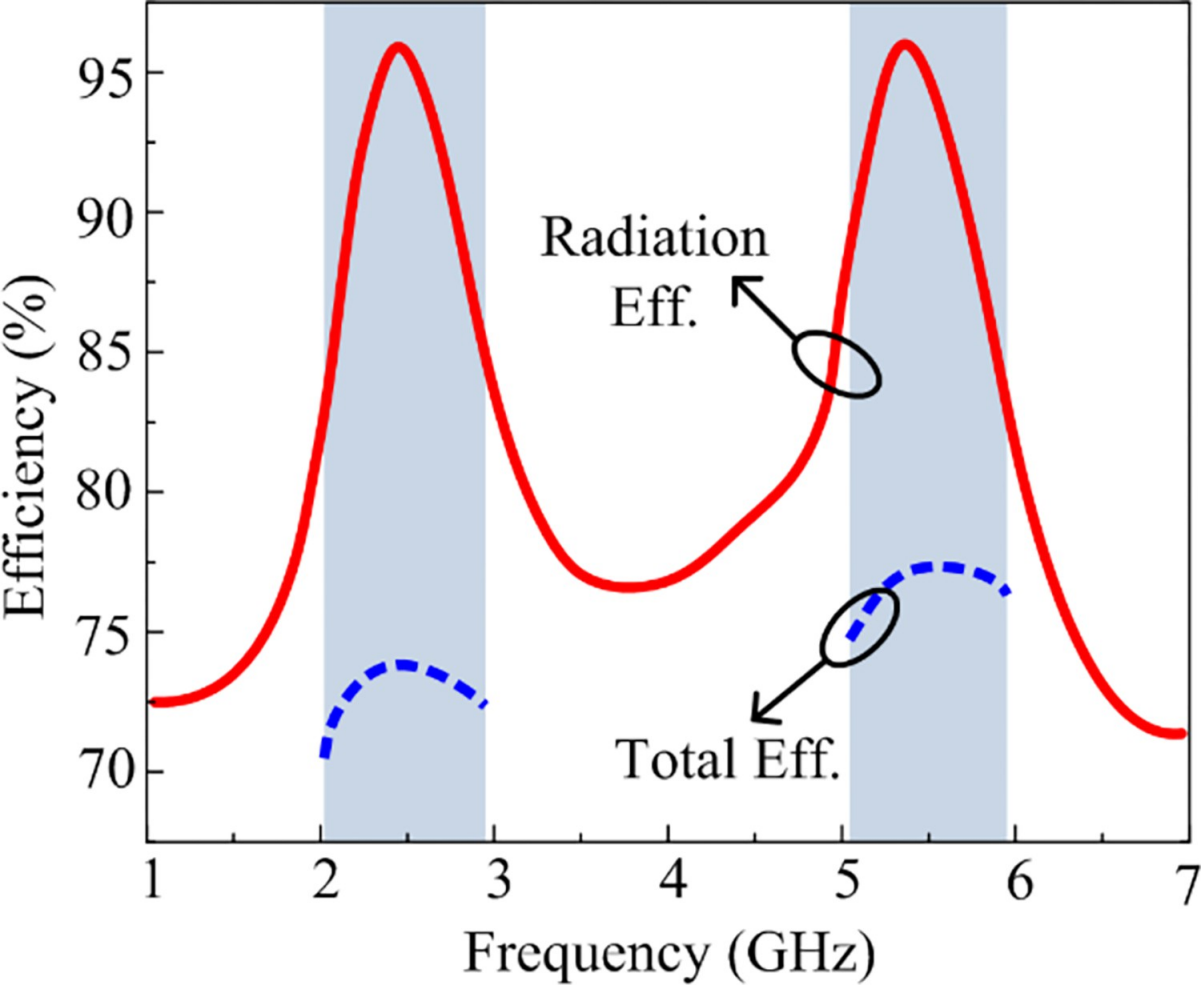
Predicted efficiency of the recommended antenna.

Fig 9 demonstrates the recommended antenna’s radiation performance in a dual-band operation. The curve below shows that the antenna operates at 2.1–2.95 GHz and 5.1–5.9 GHz with a radiation efficiency of >92%. The antenna’s maximum radiation efficiency is 96.5% at 2.45 GHz and 95% at 5.4 GHz, respectively. Moreover, the total efficiency of the antenna is also higher than 70%, which is acceptable for practical applications. Future devices using the ISM and WLAN frequency bands may profit from the suggested antenna’s higher radiation characteristics.

### Comparison with literature work

Table 2 presents a comparison between the recommended design and designs already presented in the published works. The performance analogy of the demonstrated design with the previous works is performed in terms of size, functional bandwidth, resonance frequency, and peak gain. The table demonstrates that the recommended antenna has a significant gain, a modest size, and functions over a wideband while having a simpler geometry.

**Table 2:** Comparison with literature work targeting similar bands.

Ref	Antenna Size (λ_L_ × λ_L_ × λ_L_)	Bandwidth (GHz)	Operating Frequency (GHz)	Peak Gain (dBi)
[24]	0.21 × 0.18 × 0.007	2.4–2.63/5.13–5.8	2.5/5.2	0.8/1.25
[25]	0.39 × 0.31 × 0.13	2.43–2.5	2.46	-
[26]	0.22 × 0.3 × 0.011	2.37–2.59/4.85–6.02	2.45/5.5	4/7
[27]	0.82 × 0.82 × 0.007	2.4–2.61/4.95–5.35	2.5/5.1	4.89/5.75
[28]	0.36 × 0.36 × 0.13	2.36–2.6/5.66–5.9	2.45/5.8	4.92/4.76
[29]	0.15 × 0.22 × 0.033	2.4–2.49/5.14–5.417	2.45/5.2	4.1/1.4
This Work	0.15 × 0.26 × 0.006	2.1–2.95 /5.1–5.9	2.45/5.4	4.25/5.75

## Enhanced isolation MIMO antenna design

### MIMO design methodology

**Fig 10:**
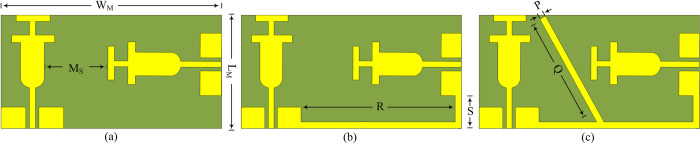
Proposed MIMO configuration (a) step-1 (b) step-2 (c) final design.

**Fig 11:**
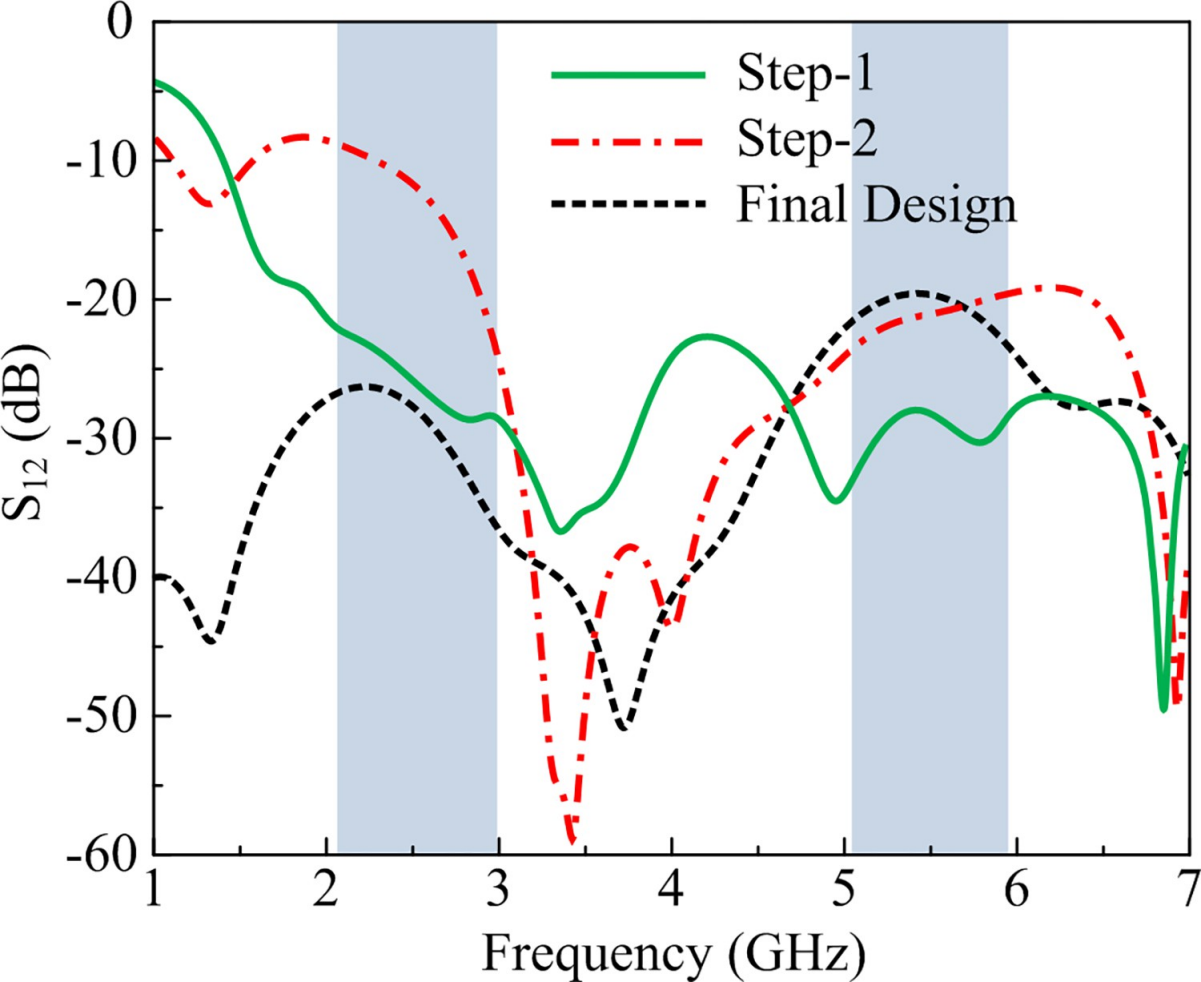
|S12/21| of proposed MIMO antenna against with and without decoupling structure.

The 2-port MIMO configuration of the proposed design given in Fig 10(A)–10(C), is adopted by placing elements of the antenna perpendicular to each other. The separation between two radiating components of the antenna is given by M_S_ = 20 mm and the dimensions of the two–port MIMO antenna is W_M_ × L_M_ = 66 mm × 33 mm. As illustrated in Fig 10(A), the step-1 offers <–25 dB at operational bandwidth of 2.1–2.95 GHz and <–18 dB at an operational bandwidth of 5.1–5.9 GHz, as depicted in Fig 11. However, due to the lack of a common ground plane the practical usage will be very limited, so the step-2 is performed by connecting the ground plane, as depicted in Fig 10(B). This step results in high mutual coupling across the lower resonance, as shown in Fig 11. Thus, to achieve the acceptable level of mutual coupling which is recommended to be less than –20 dB, step-3 is performed by loading an additional open-ended stub to the ground as shown in Fig 10(C). The mutual coupling of the antenna is lowered to < –22 dB and <–27 dB and respective lower and higher resonance, as depicted in Fig 11.

### Fabrication and measurement setup

**Fig 12:**
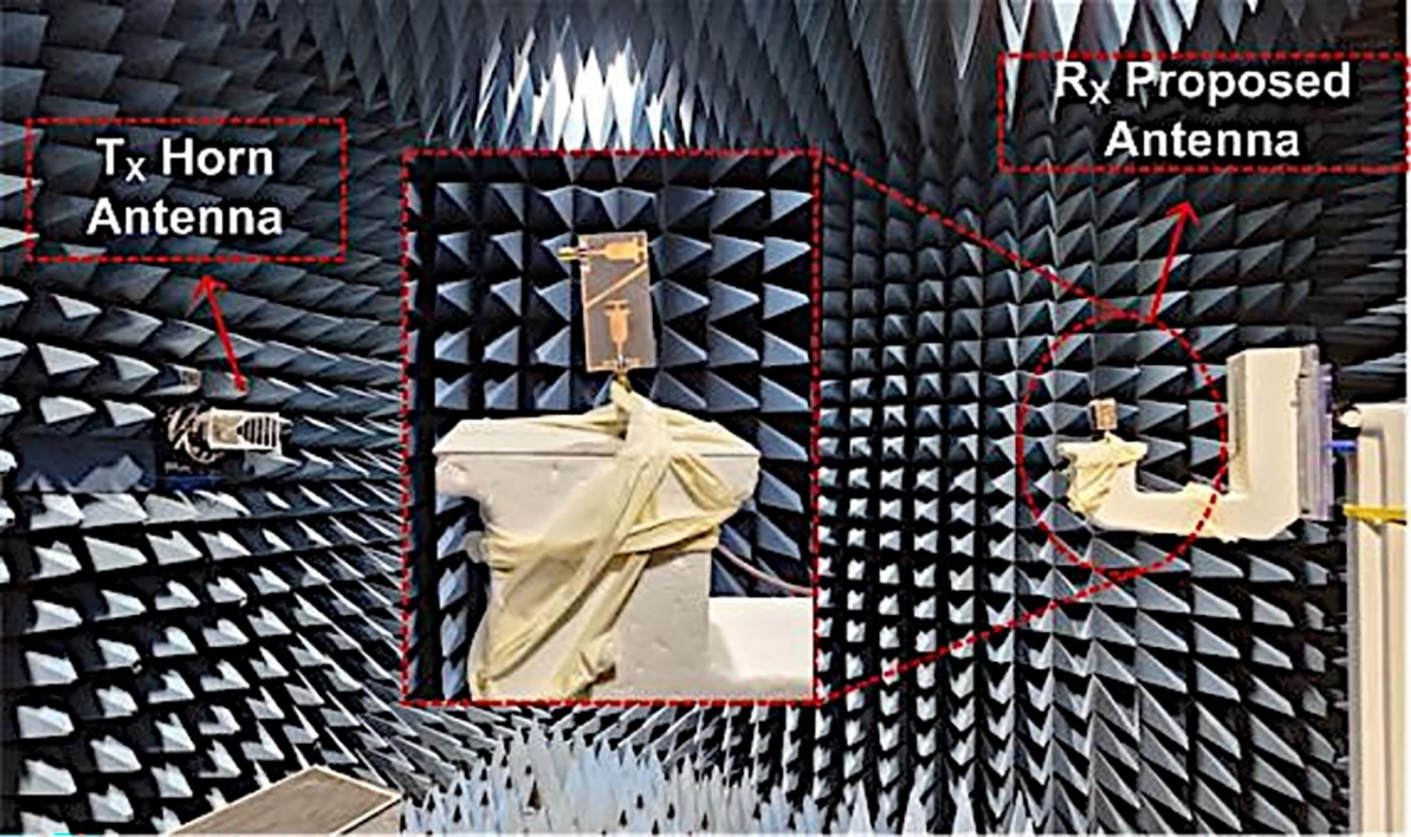
Far-field measurement of proposed dual band two port MIMO antenna.

Fig 12 demonstrates the implemented prototype of the structure along with the testing equipment. With a gold-plated SMA connector with a 50-ohm impedance, the antenna is stimulated. The reflection coefficient of the suggested dual-band antenna is investigated and verified by using a Vector Network Analyzer (VNA). The far-field results are measured by placing the developed prototype in front of the Horn antenna as a reference and having a broadband range of 12 GHz within an anechoic chamber.

## Results of the proposed MIMO configuration

This section discusses the performance parameters (coupling, radiation pattern, ECC, CCL, DG, and MEG) of the suggested two-port MIMO system using the common ground technique used for isolation improvement. The performance parameters in the form of reflection and transmission coefficient, gain and radiation pattern as well as MIMO parameters are examined.

### Reflection coefficient

**Fig 13:**
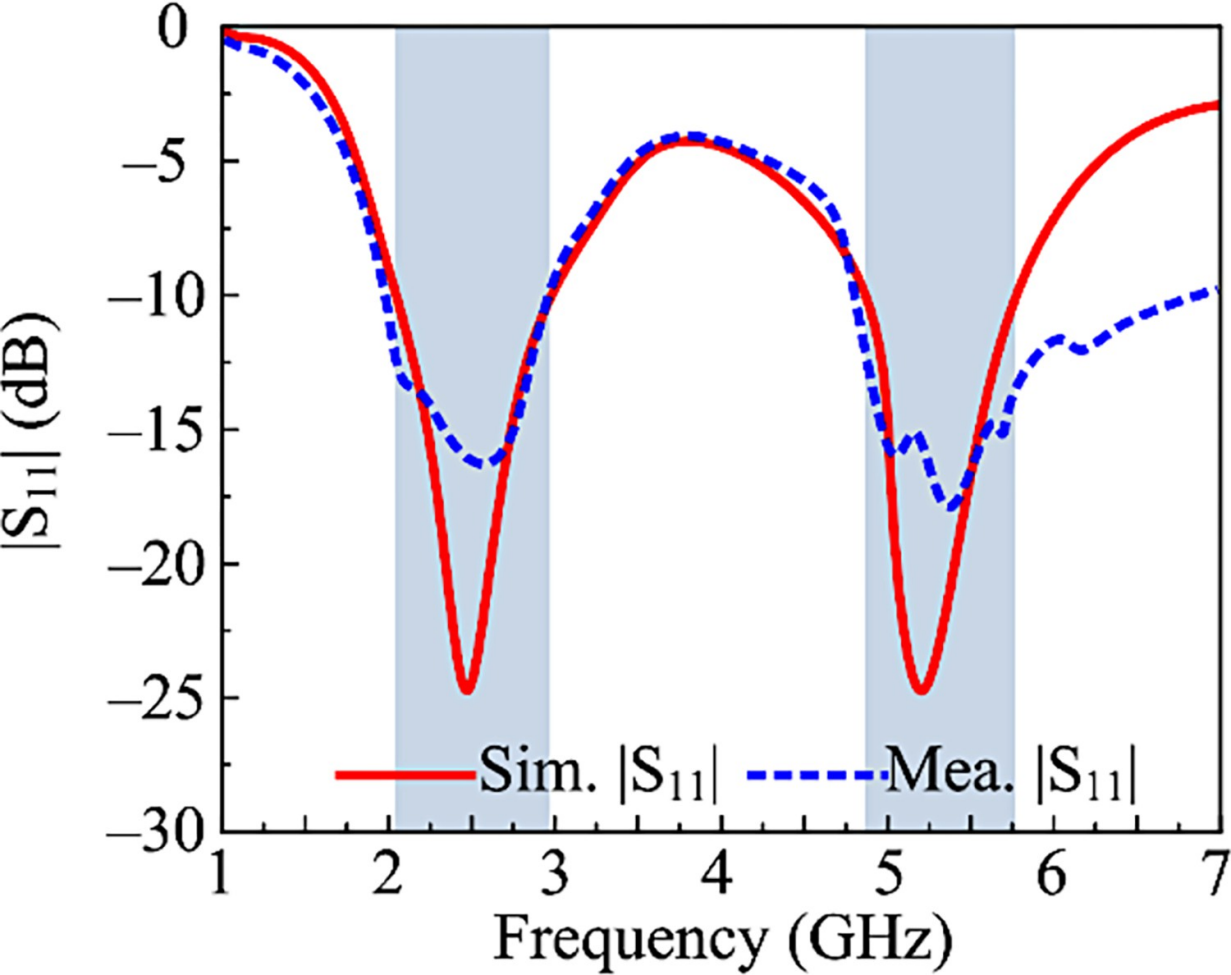
Predicated and hardware-tested reflection coefficient of recommended 2-port MIMO configuration with the proposed coupling reduction techniques.

The designed double band, two-port MIMO antenna measured and resulted reflection S-Parameters are given in Fig 13. The recommended MIMO antenna, which operates at the dual frequencies of 2.45 GHz and 5.4 GHz, has an impedance bandwidth of (|S11| > -10) 2.1–2.95 GHz and 5.12–5.9 GHz, in accordance with the figure. Also, the measurements and software findings exhibit striking similarities, making the suggested architecture the best applicant for upcoming 5G wireless devices that operate over the ISM and WLAN frequency ranges.

### Transmission coefficient

**Fig 14:**
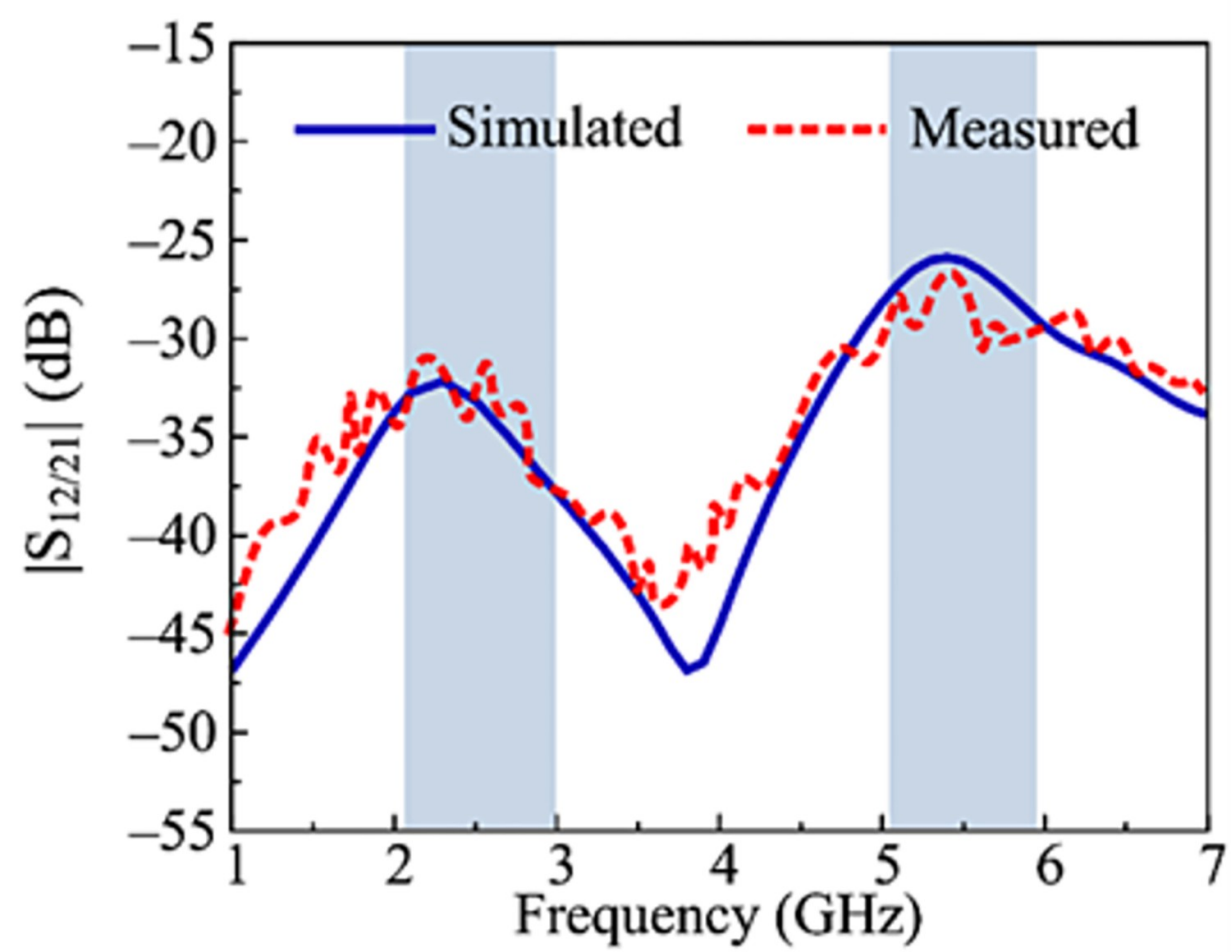
Predicated and hardware tested transmission coefficient of recommended two port MIMO antenna with decoupling structure.

The transmission coefficient of the recommended two-port MIMO antenna is illustrated in Fig 14. The isolation of antenna can be examined by studying transmission co-efficient. It can be observed that antenna yields |S21| and |S12| less than– 30 dB at operational band of 2.45 GHz and |S21| and |S21| less than– 27 dB at working bandwidth of 5.4 GHz. Moreover, the results also show the resemblance in predicted and tested outcomes, which pressure recommended antenna a prospective choice for upcoming technology working over 2.45 GHz and 5.4 GHz.

### Peak gain

**Fig 15:**
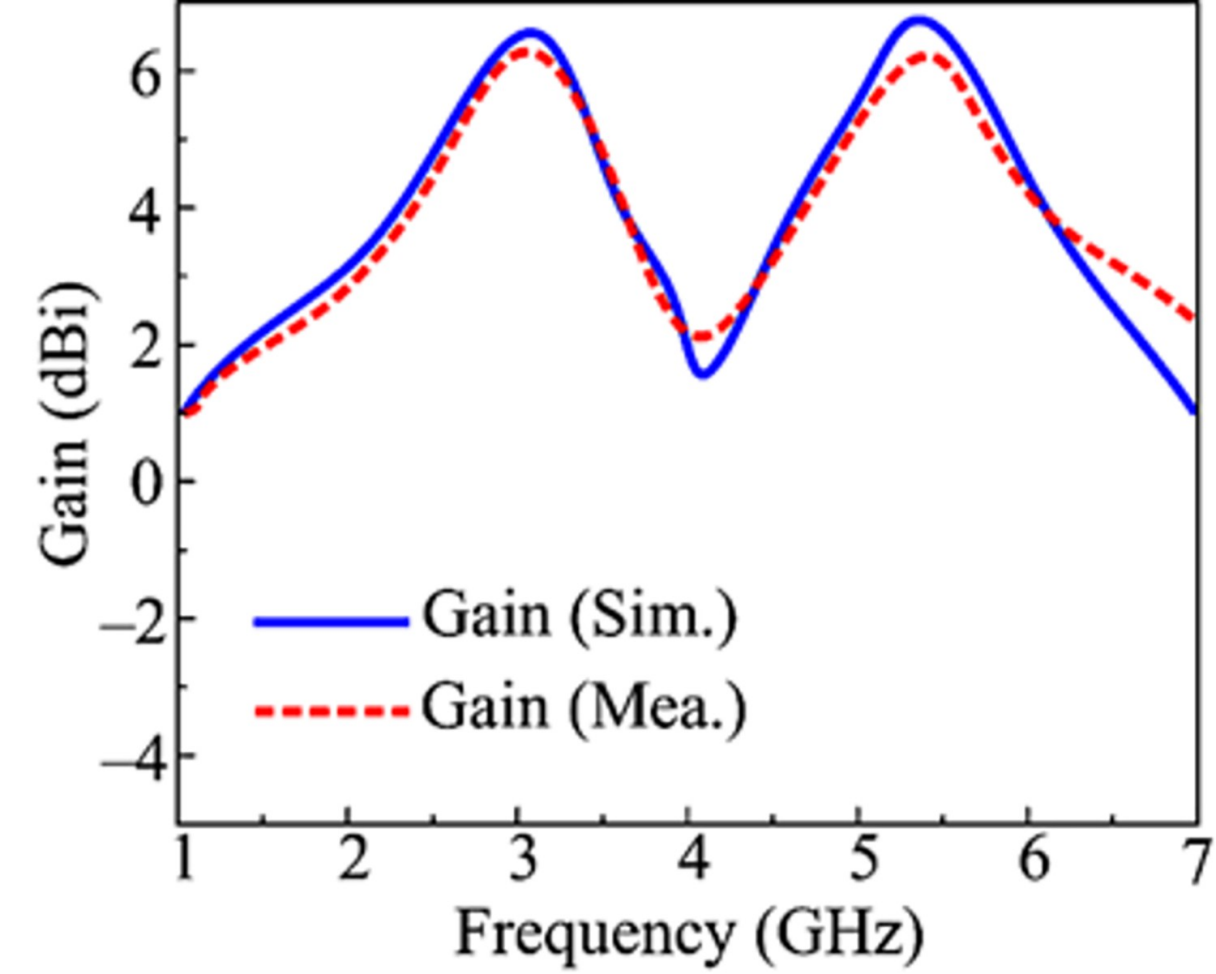
Predicated and measured gain of proposed work.

The projected and actual gains of the suggested design are displayed in Fig 15. With a high value of 6.25 dBi at 2.45 GHz, the proposed 2-port MIMO antenna literally gives a gain > 6 dBi over the operational bandwidth of 2.1–2.95 GHz. The antenna, on the other side, delivers a gain of > 6.25 dBi in the operative 5.1–5.9 GHz range and a maximum value of 6.4 dBi at the resonant frequencies of 5.4 GHz. The graphic demonstrates that the differences between measured and simulated outcomes are generally small. The gain provided and the parallels in measured and simulated gains make the recommended antenna a strong contender for future tiny devices operating over high gain.

### Radiation pattern

**Fig 16:**
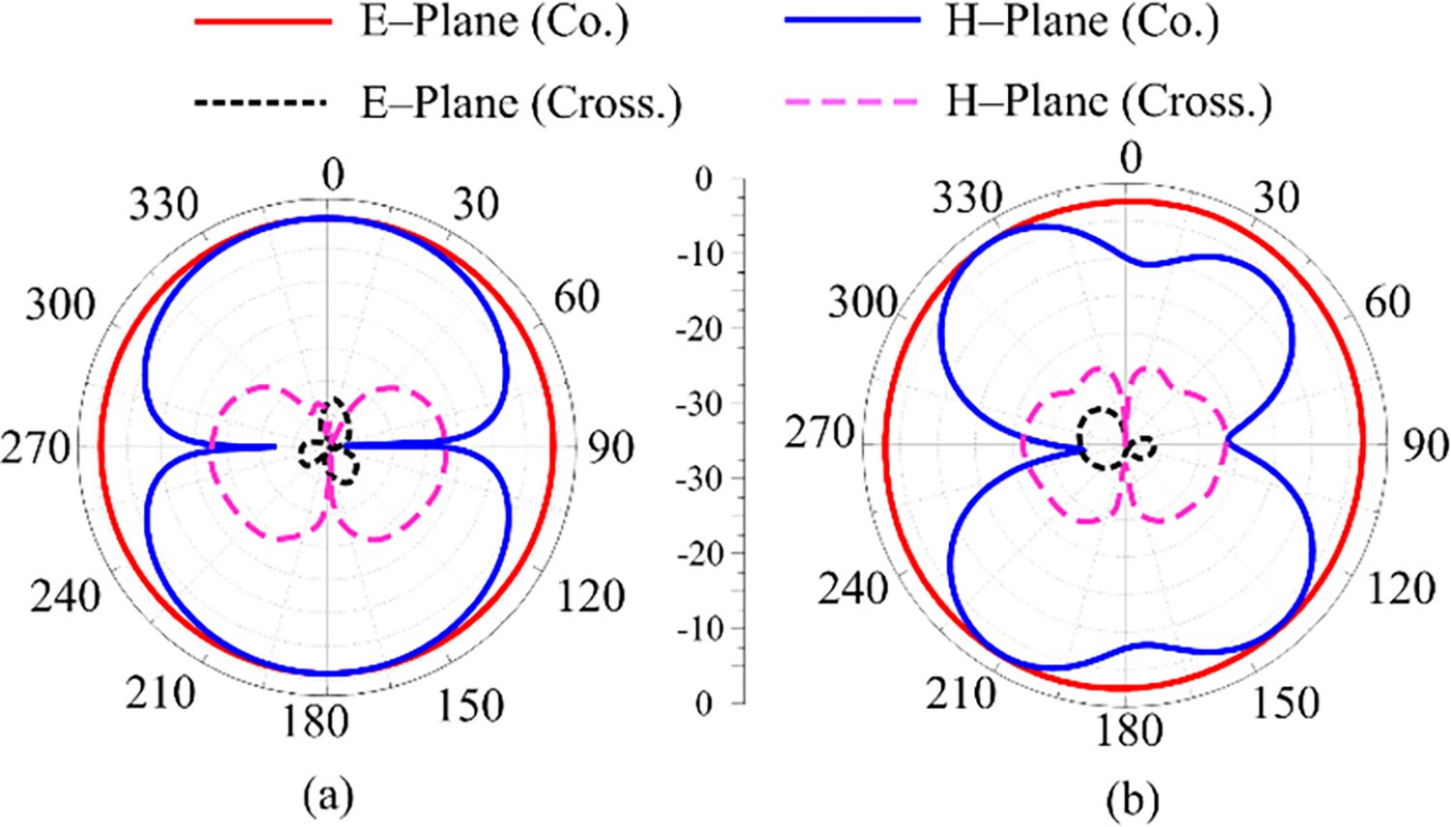
Simulated co- and cross polarization of the proposed two port MIMO antenna with decoupling structure at (a) 2.45 GHz (b) 5.4 GHz.

Fig 16(A) and 16(B) reveals the suggested two-port antenna’s radiation pattern at 2.45 GHz and 5.4 GHz, respectively. It can be examined that the suggested work delivers an omnidirectional radiation behavior in the E-plane and a bi-directional radiation in the H-plane at a resonance frequency of 2.45 GHz. For 5.4 GHz, the E–plane pattern is same as 2.45 GHz, which is omnidirectional but for H–plane the pattern shows small distortion. This distortion is due to the multi–stub loading and shifting towards higher frequency. Moreover, low cross polarization is observed in both planes at 2.45 GHz and 5.8 GHz having maxi-mum cross-polarization of -20 dB.

### Envelope correlation coefficient

**Fig 17:**
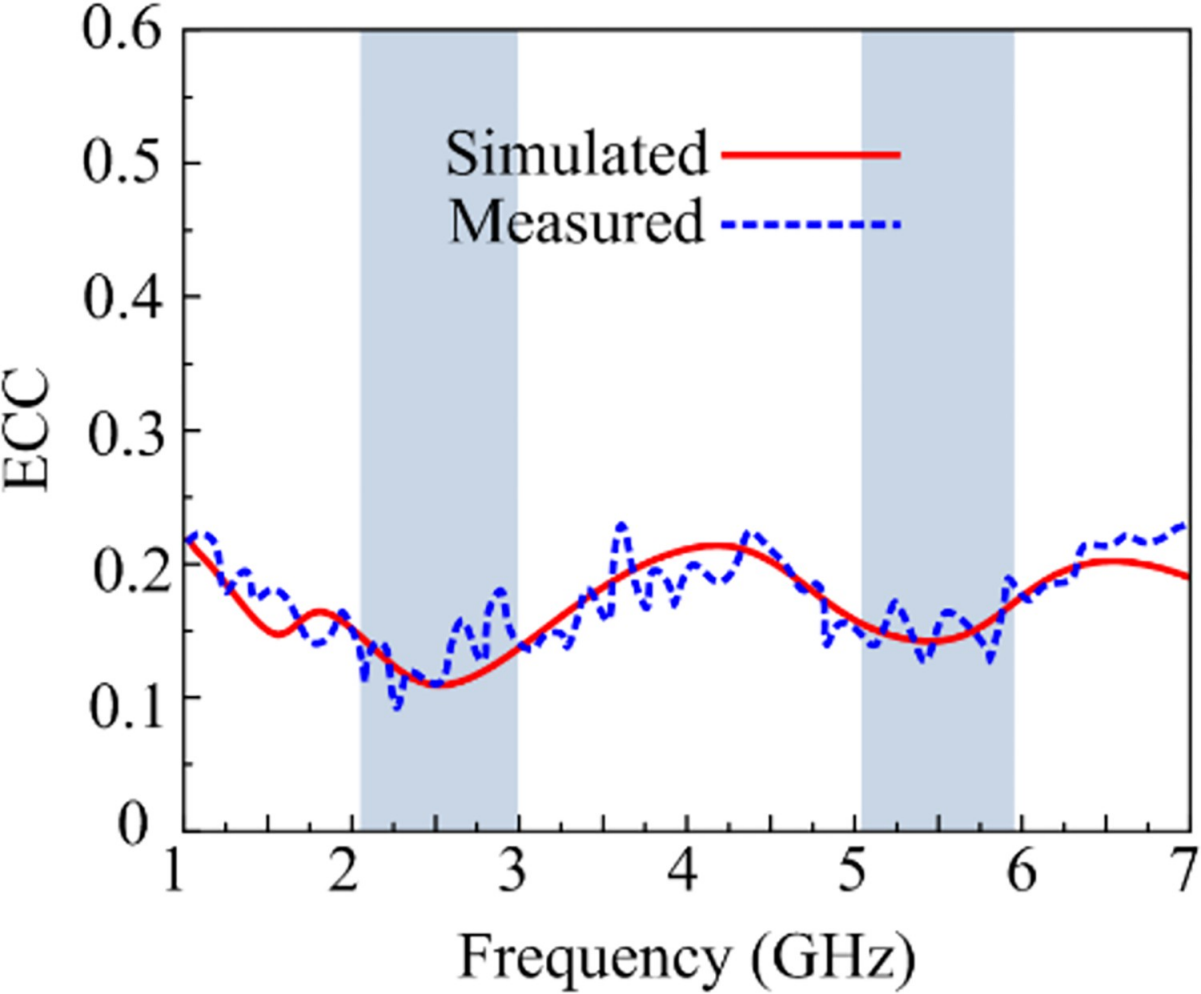
ECC of suggested work.

Because the MIMO system combines many antenna components, mutual coupling becomes crucial owing to antenna positioning. Fig 17 displays the ECC for the recommended MIMO antenna. For said ISM and WLAN operating frequency bands, the 2-port MIMO configuration that is recommended has an EEC value of 0.2, ensuring independent channel operation and the greatest diversity performance. ECC should be less than 0.5, which is considered acceptable [47].

### Channel capacity loss

**Fig 18:**
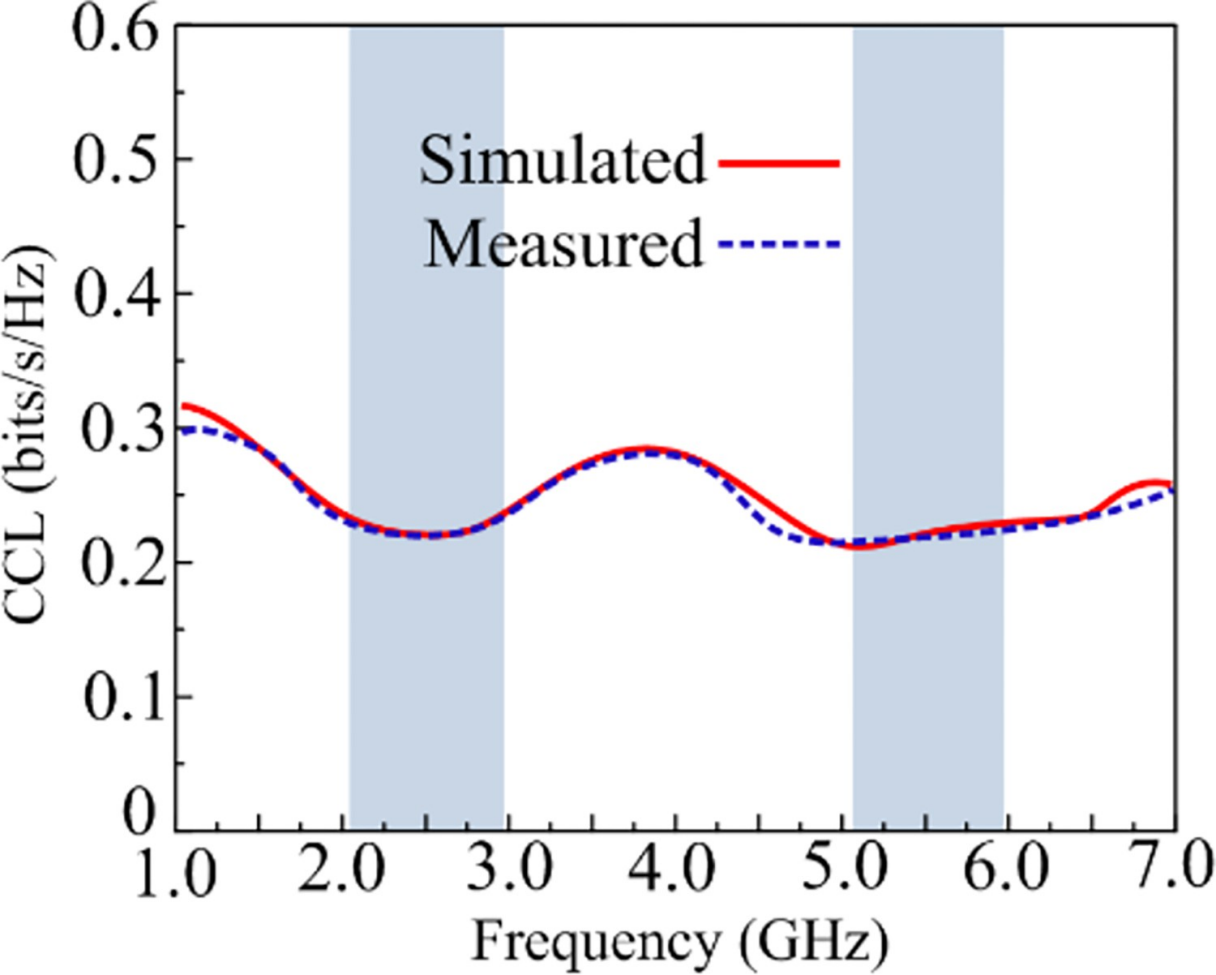
CCL performance of suggested work.

Another dominant characteristic for MIMO antennas is CCL, which indicates a re-duction in MIMO capacity as a result of correlation in MIMO connections. The CCL curves for the recommended MIMO antenna are mentioned in Fig 18. The Figure represents that a CLL of 0.21 bits/Hz/sec at an operating bandwidth of 5.1–5.9 GHz and 0.22 bits/Hz/sec at an operational bandwidth of 2.1–2.95 GHz is offered by the antenna. The CLL value must be less than 0.6 bits/Hz/sec [48].

### Diversity gain

**Fig 19:**
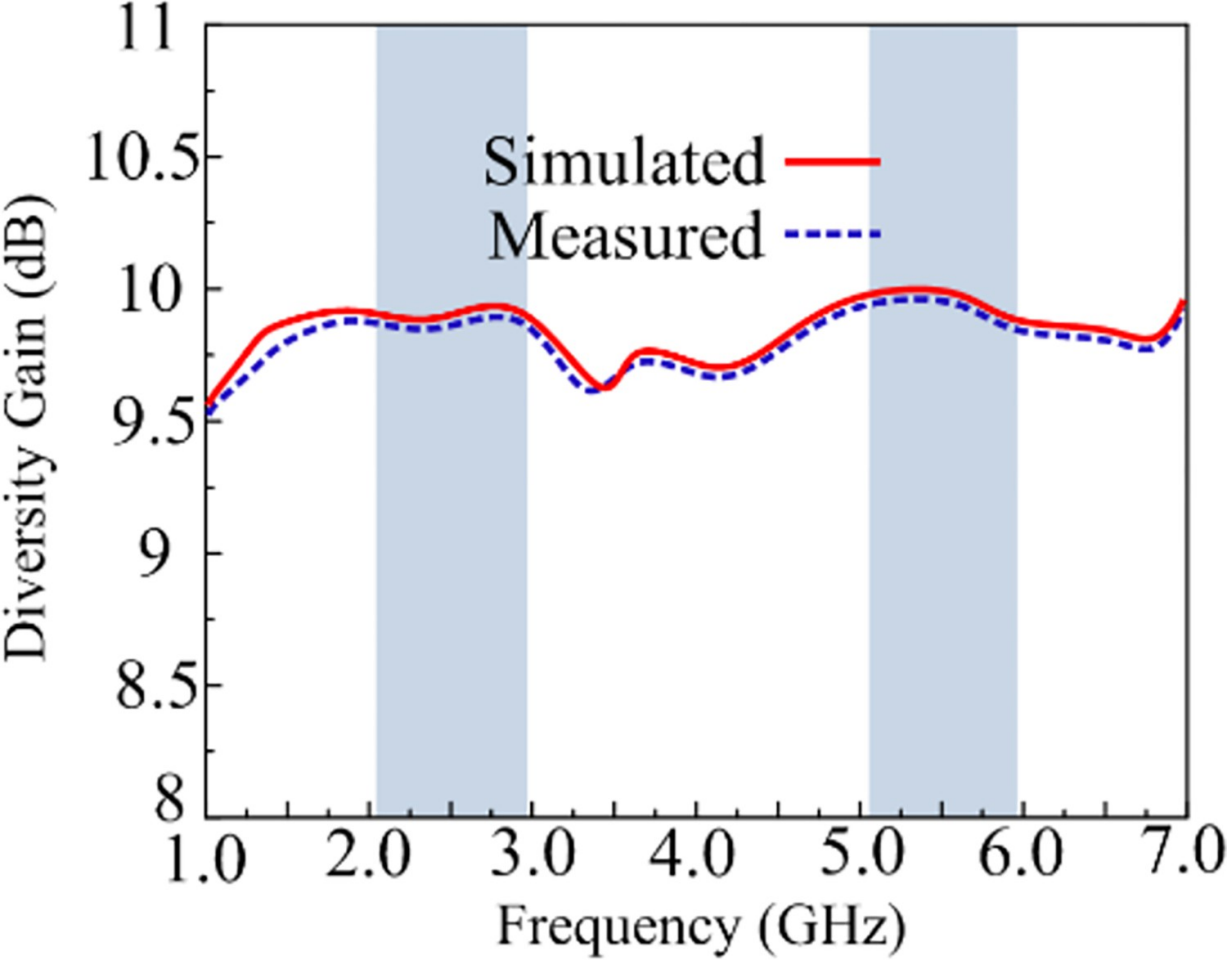
DG of suggested work.

DG, which estimates the magnitude of transmission power drop that follows from employing a diversity scheme without impacting the performance of the MIMO antennas, is yet another significant performance metric. A DG around 9.99 dB is offered by the suggested MIMO antenna design at the operational bandwidths of 2.1–2.95 GHz and 5.1–5.9 GHz, as shown in Fig 19. The ideal value for the DG and the appropriate antenna is both about 10 dB [49].

### Mean effective gain

**Fig 20:**
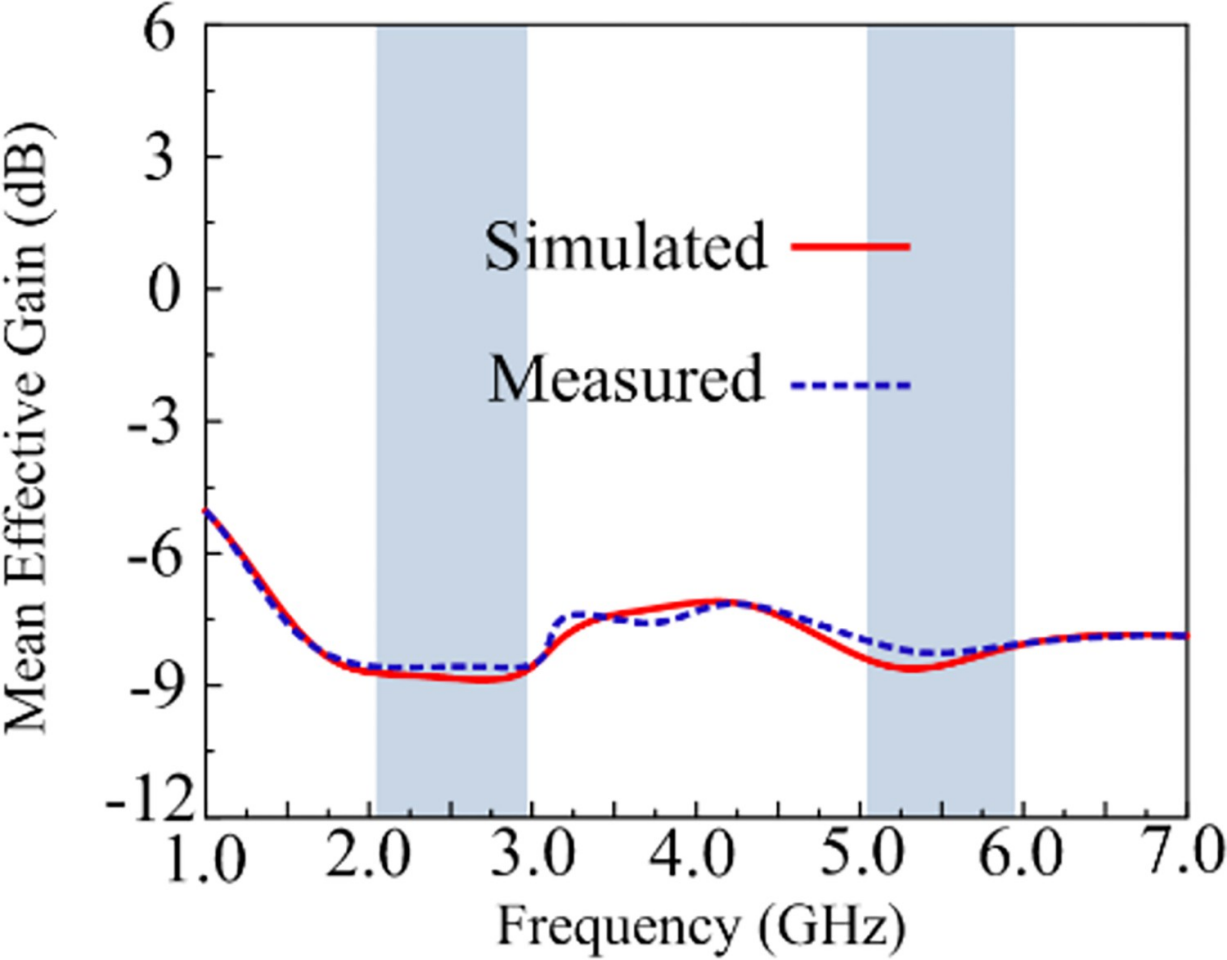
MEG of suggested work.

MEG is a further criterion for evaluating MIMO antenna performance. It speaks about the power that a wireless system receives in a fading environment. MEG should not exceed a value of -3 dB [50]. Fig 20 shows that the demonstrated two-port MIMO antenna’s MEG is within an acceptable range at -8 dB.

### SAR analysis

**Fig 21:**
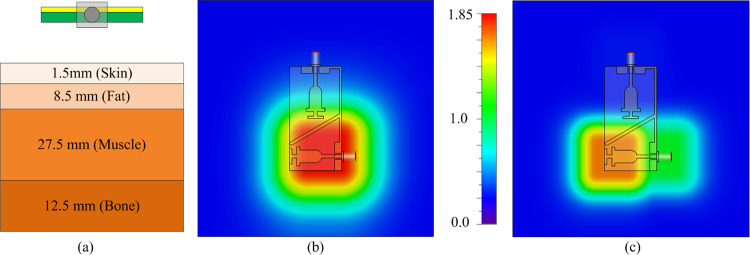
SAR analysis (a) simulation setup; simulated results at (b) 2.45 GHz (c) 5.8 GHz.

As the demonstrated antenna might be used near human body applications in telemetry equipment used for monitoring patients in hospital rooms or intensive care units, the SAR analysis is a critically important parameter to investigate. The SAR analysis is carried out using the 4-layer equivalent model of the human body consisting of skin, fat, muscle, and bone, as depicted in Fig 21(A). The MIMO antenna is placed at a gap of 10mm from the nearest layer of skin. The permittivity and conductivity at 2.4 GHz are set to be 38.06 and 1.44 for skin, 5.28 and 0.1 for fat, 52.79 and 1.7 for muscle, 11.41 and 0.38459 for bone, respectively [28]. Likewise, at 5.8 the permittivity values for skin, fat, muscle and bone are 35.114, 4.9549, 48.485, and 9.6744 while the conductivity values are set to be 3.71, 0.29, 4.96, and 1.544, respectively [51]. The SAR analysis was carried out initially using the input power of 1W, thereafter the value was decreased to see the input power limit, Fig 21(B) and 21(C) shows the simulated results of antenna for SAR analysis at both resonating frequencies. The analysis shows that the antenna offers SAR level of 1.37 and 1.81 W/kg respectively, over 1g and 10g tissues, at 2.4 GHz with the input power of 200mW. Likewise, for 5.8 GHz the SAR values of 1.12 W/kg and 1.59 W/kg are observed over the 1g and 10g tissues. Thus, the findings indicate that the suggested MIMO antenna having a maximum input power of 200mW offers the SAR values within the limit of the standard of 1.6 W/kg over 1g and 2 W/kg over 10 g tissue, set by FCC and the European Union.

### Comparison with MIMO works

Table 3 shows the comparison of suggested isolation improved MIMO antenna by using the common ground plane with literature work. The comparison is performed in respect of antenna size, number of elements in the antenna, ECC, and the achieved mutual coupling. The technique used to minimize mutual coupling is also listed. The table shows that the suggested antenna has small size and low coupling along with wide operational bandwidth. Thus, the proposed MIMO antenna is a promising candidate for future com-pact wireless devices operating over dual band and high gain.

**Table 3:** Performance results of suggested work along with literary works.

Ref	Antenna Size (λ_L_ × λ_L_)	Bandwidth (GHz)	No. of Port	ECC	Mutual coupling (dB)	Max. Gain (dB)	Common Ground Plane
[32]	0.33 × 0.33	2.1–2.7/5.1–6.1	2	0.15	-18	4.4	Yes
[33]	0.36 × 0.25	2.29–2.47/4.57–6.75	2	0.003	-22	4.3	Yes
[38]	0.82 × 0.82	2.5–2.55	2	-	-20	6.1	Yes
[40]	0.4 × 0.33	2.12–2.8/4.95–6.65	2	0.3	-15	6.19	Yes
[41]	0.26 × 0.26	2.36–2.59/3.17–3.77	2	0.02	-15	5.9	Yes
This Work	0.26 × 0.53	2.1–2.95/5.1–5.9	2	0.2	-22	6.25	Yes

## Conclusions

In this manuscript, a simplified geometry, compact size and low–profile antenna for ISM and WLAN application is proposed. The antenna offers wideband of 2.1–2.95 GHz and 5.1–5.9 GHz respectively at the functioning frequencies band of 2.45 GHz and 5.4 GHz. Later on, two–port MIMO configuration was adopted to make it able for future wire-less communication. The two–port MIMO antenna offers mutual coupling value of around –18 dB which is not in acceptable range. To improve mutual coupling, a common ground technique along with a stub is utilized to obtain a lower mutual coupling of –27 dB. The antenna offers a peak gain of 6.25 dBi and ECC around 0.2. The performance results obtained by EM software are also compared with hardware prototype results, which show quite similarities. Moreover, the performance of the suggested MIMO and single-element design is contrasted with previously published works in the field. The results and performance parameters make the recommended antenna a prospective choice for wireless systems performing at the ISM and WLAN band spectrums because of the characteristics of high gain and broad bandwidth and having compact size along with simplified geometry.
