# Correction: Dual-band MIMO antenna with low mutual coupling for 2.4/5.8 GHz communication and wearable technologies

**DOI:** 10.1371/journal.pone.0305186

**Published:** 2024-06-04

**Authors:** Wahaj Abbas Awan, Tanvir Islam, Fahad N. Alsunaydih, Fahd Alsaleem, Khaled Alhassoon

The fifth author’s name is spelled incorrectly. The correct name is Khaled Alhassoon and correct citation is: Awan WA, Islam T, N. Alsunaydih F, Alsaleem F, Alhassoon K (2024) Dual-band MIMO antenna with low mutual coupling for 2.4/5.8 GHz communication and wearable technologies. PLoS ONE 19(4): e0301924. https://doi.org/10.1371/journal.pone.0301924.

The funding statement is incorrect. The correct funding statement is: The Researchers would like to thank the Deanship of Graduate Studies and Scientific Research at Qassim University for financial support (QU-APC-2024-9/1).
